# Comparative Invitro Testing of the Tensile Bond Strength Under Artificial Aging Between Different Lithium Disilicate Ceramics to Composite Substrate: A Novel Methodology

**DOI:** 10.7759/cureus.66163

**Published:** 2024-08-05

**Authors:** Osamah A Alsulimani, Abdulrahaman J Alhaddad, Arwa U AlSaggaf, Mosa Altassan, Mazen Alghamdi, Samar H Abuzinadah, Maher S Hajjaj, Amin A Marghalani

**Affiliations:** 1 Department of Oral Diagnostic Sciences, Faculty of Dentistry, King Abdulaziz University, Jeddah, SAU; 2 Department of Oral and Maxillofacial Prosthodontics, Faculty of Dentistry, King Abdulaziz University, Jeddah, SAU; 3 Department of Oral and Maxillofacial Surgery, College of Dental Medicine, Umm Al-Qura University, Makkah, SAU; 4 Department of General Dentistry, Seven Stars Clinic, Jeddah, SAU; 5 Department of Restorative Dentistry, Faculty of Dentistry, King Abdulaziz University, Jeddah, SAU

**Keywords:** tensile strength, thermocycling, lithium disilicate, initial lisi, ips e.max

## Abstract

Objective

The purpose of this study is to compare the tensile bond strength values to composite substrate pre- and post-aging between IPS E.max CAD and Initial LiSi.

Methods

The study utilized four blocks of IPS E.max CAD LT/B1 C14 (Ivoclar Vivadent, Liechtenstein, Germany) (referred to as E) and four blocks of Initial LiSi LT/B1 (GC, Tokyo, Japan) (referred to as L). These blocks were milled to produce 76 ceramic bars measuring 2 mm × 2 mm × 10 mm (E = 38, L = 38/n = 19). After acid etching with hydrofluoric acid (BISCO, Schaumburg, IL, USA) and silane application (BIS-SILANE, BISCO), the specimens were embedded in putty (Express STD, 3M, Decatur, AL, USA) to create a mold for the resin cement (RelyX U200, 3M). Subsequently, one group of each brand underwent mechanical tensile testing (E0 and L0), while the other groups were subject to tensile testing after artificial aging involving 500 thermal cycles between 5 and 55°C (E5 and L5). The mean tensile strength for each group (E0, E5, L0, and L5) was determined using the Brown-Forsythe one-way ANOVA and Tamhane’s post hoc tests.

Results

Initial LiSi showed a superior pre-aging mean (11.7 MPa). However, both materials had identical post-aging means (7.6 MPa). There were no statistically significant differences, except between the dependent Initial LiSi groups (L0-L5). Most failure modes were mixed (cohesive cement and adhesive). There were no cohesive failures on the cement side except in three specimens of Initial LiSi post-aging.

Conclusion

The tested conditions have shown that Initial Lisi exhibited the highest pre-aging mean; however, it exhibited inferior bond stability under aging conditions compared to IPS E.max CAD. Analyzing the microstructure before and after aging may provide insights into the greater decrease in bond strength observed in the Initial LiSi specimens.

## Introduction

In dentistry, ceramics are frequently employed because of their pleasing appearance, high flexural strength, and good cumulative survival rates [1]. The properties they possess are beneficial, such as chemical stability, compatibility with living tissues, poor heat transfer, high resistance to compression, ability to spread heat, transparency, luminescence, and a similar thermal expansion rate as teeth [2]. Dental ceramics are generally categorized into glass and crystalline ceramics based on their composition. Glass ceramics have advantages over many other restorative materials because they exhibit high bonding strength to the structure and can be used as veneers and crowns with good esthetics due to the optical properties emitted by the predominant glass phase. Restorations may be constructed monolithically or layered with veneering porcelain for anterior and posterior single restorations.

One factor in the clinical success of glass ceramic restorations is the quality and durability of the bond between ceramic and resin cement. The quality of this bond is a factor of bonding mechanisms, which is determined in part by the method of surface conditioning aimed at creating chemical or micromechanical retention to ceramic substrate. Ceramic bonding has long been the gold standard for retaining and reinforcing low- to medium-strength silica-based ceramics. Still, multiple pretreatment steps are required for the bonding surfaces, increasing complexity and technique sensitivity compared to traditional cementation protocols. A monoblock is obtained if the bonding procedure is applied successfully [3]. For a successful bond, the surfaces of the parts to be bonded should be thoroughly conditioned to get a good junction between the molecules of the bond and the workpieces. This link must be strong enough to withstand stresses within the bonding agent that are generated by polymerization and shrinkage [4]. Acid etching with hydrofluoric (HF) acid has been proven to be the optimum surface treatment protocol for glass ceramics [5]. The glassy matrix is selectively removed, and crystalline structures are exposed. HF solutions between 2.5% and 10% applied for two to three minutes seem the most successful. The amount, size, and distribution of lithium disilicate crystals influence the formation of microporosities that acid etching creates [6-9].

Lithium disilicate glass-ceramic is a commonly used restoration for indirect single units. Its high tensile bond values distinguish it due to the amount of glass content; this expands its application when more adhesion is needed, particularly in partial coverage restorations. IPS E.max CAD (Ivoclar Vivadent, Liechtenstein, Germany) is a renowned lithium disilicate model. The material gained traction after its launch in 2006, prepared explicitly for CAD/CAM soft milling; the material comes prepared during a “blue state,” allowing easier machining and intraoral occlusal adjustment [10]. In 2016, GC America introduced a new lithium silicate/disilicate CAD/CAM glass-ceramic model into the dental market, named Initial LiSi [11]. Initial LiSi is a fully crystallized lithium disilicate that delivers optimal physical and aesthetic properties [12]. The flexural strength of LiSi and E.max lithium disilicate is relatively similar, at 500 MPa and 400, respectively [13]. On the microstructure, Initial LiSi exhibits different sizes and distributions of lithium disilicate crystals (1-1.4 μm) and is more densely distributed than IPS E.max CAD, which exhibits many larger lithium disilicate crystals up to 1-4 μm [14]. So far, the literature has shown that Initial LiSi demonstrates relatively similar physical and chemical properties compared to IPS E.max [15].

Studies assessing lithium disilicate restorations have shown high clinical performance and debonding resistance. RelyX U200 is a self-adhesive dual-cure resin cement containing acids and hydrophilic monomers. It is suitable for various applications, including all ceramic systems, as per the manufacturer’s recommendations. IPS E.max is the most used glass-ceramic among the lithium disilicate category, with a plethora of evidence in the literature [16-18]. Since Initial LiSi is still considered new in the dental market, clinical and laboratory evidence is scarce. This research aims to determine which material is superior in tensile bond strength (TBS) under simulated aging conditions. The significance of this study lies in its potential to contribute to the understanding of the performance and longevity of dental restorations made from lithium disilicate glass-ceramics. The null hypotheses are as follows: (i) there is no statistically significant difference between the tensile strength means of IPS E.max CAD and Initial LiSi, and (ii) there is no statistically significant difference between the pre- and post-aging tensile strength means of IPS E.max CAD and Initial LiSi.

## Materials and methods

Materials

Two materials were selected: IPS E.max CAD LT/B1 C14 (Ivoclar Vivadent, Liechtenstein, Germany) and Initial LISI LT/B1 (GC, Tokyo, Japan). Table 1 contains the mechanical properties as provided by the manufacturer.

**Table 1:** Composition and mechanical properties of the tested materials

	IPS E.max CAD	Initial LiSi
Composition	Lithium disilicate crystals 70% Li_2_Si_2_O_5_ embedded in a glassy matrix	Lithium disilicate micro-crystals equally dispersed in a glass matrix
Density (g/cm^2^)	2.5	2.4
Crystal system	Lithium disilicate crystals measure 3-6 µm in length	Lithium disilicate crystals measure 1.5 × 0.5 µm
Compressive strength (MPa)	433	454
Fracture toughness Kıᴄ (MPa/m^2^)	2.13	1.51
Thermal expansion coefficient (×10^-6^/K)	10.55 ± 0.35 10^-6^/K	9.8 × 10^-6^/K
Glass transition temperature (C)	560	520

Specimen preparation

All specimens were prepared to the planned dimensions at 25°C. Eight lithium disilicate CAD blocks were used: four blocks of IPS E.max (E) and four blocks of Initial LiSi (L). Blocks were milled to give 76 ceramic bars (2 × 2 × 10 mm) (E = 38, L = 38/n = 19); the dimension recommended by the ISO guidelines couldn’t be applied as the block dimension is smaller. The milling process was done by Ceramill Motion 2 milling machines (Amann Girrbach, Pforzheim, Germany), Ceramill Mind 2 v25.4_7545/6 designing software, and diamond RFID blades size of 0.4, 1.0, 1.4, and 1.8 mm.

Initial LiSi blocks are provided fully sintered; thereby, no post-sintering is needed. IPS E.max blocks (supplied partially sintered) were fully crystallized after milling using the Programat P510 (Ivoclar Vivadent). The specimens were then prepared for post-glazing as follows: pre-drying phase 0-450°C over five minutes with the lid open; the lid was closed, and the firing phase started by increasing the temperature from 450 to 830°C over 12 minutes, then from 830 to 850°C over three minutes; the temperature maintained at 850°C for seven minutes; cooling phase for four minutes; lid started opening at 700°C and was open after one minute; the device beeped indicating the end of the process. The 76 ceramic bars were divided randomly and equally by convenience into four groups (E0, E5, L0, L5/n = 19).

**Figure 1:**
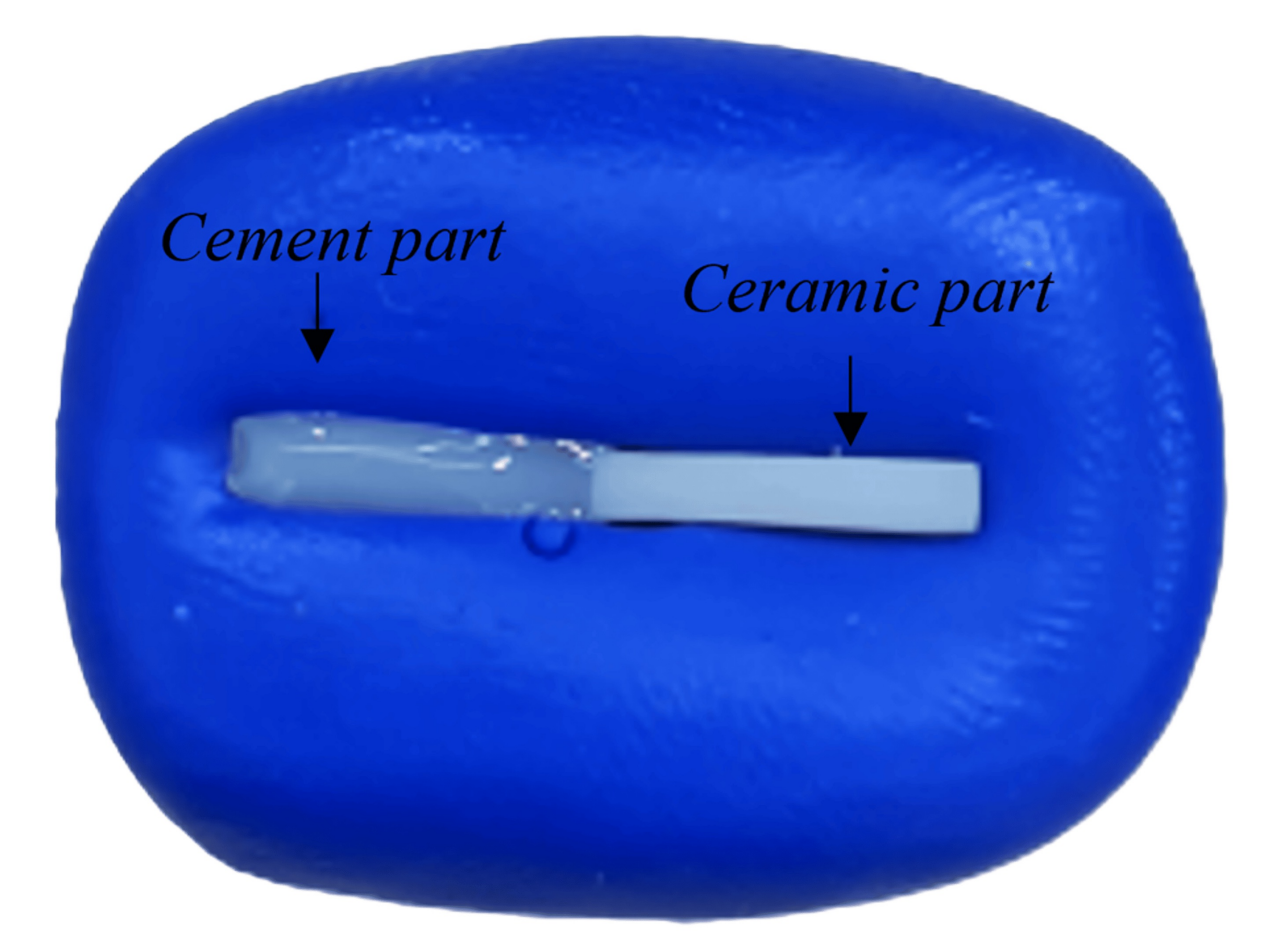
Preparation of the specimen Each specimen was fitted on one side of the putty mold, and the other side was filled with resin.

Specimens were put in a water bath (Powersonic 405) at 37°C for 24 hours. The specimens were polished with 600-800 grit sandpapers, then water sprayed and air dried. Subsequently, specimens were acid etched for one minute with HF acid (BISCO, Schaumburg, IL, USA) (9.5% HF) on the area of interest (2 × 2 mm) followed by application of one thin coat of silane (BIS-SILANE, BISCO) for one minute and then air-dried until the surface was dull. A bonding mold was created by embedding a 2 × 2 × 20 mm bar into a putty mix (Express STD, 3M, Decatur, AL, USA) until the mix was set. Each specimen was fitted on one side of the mold, and the other side was filled with resin cement (RelyX U200, 3M) incrementally to ensure complete adaptation of the cement (Figure 1). The cement was light-cured (Elipar, 3M; 600 mW/cm^2^) for 20 seconds from a 2 mm distance.

Specimens grouping 

The groups were E0, E5, L0, and L5. The letter E represents the IPS E.max CAD groups; the letter L represents the LiSi group. Groups that did not undergo thermocycling are represented by the number 0, and groups that underwent 500 cycles are represented by the number 5.

Tensile bond test

**Figure 2:**
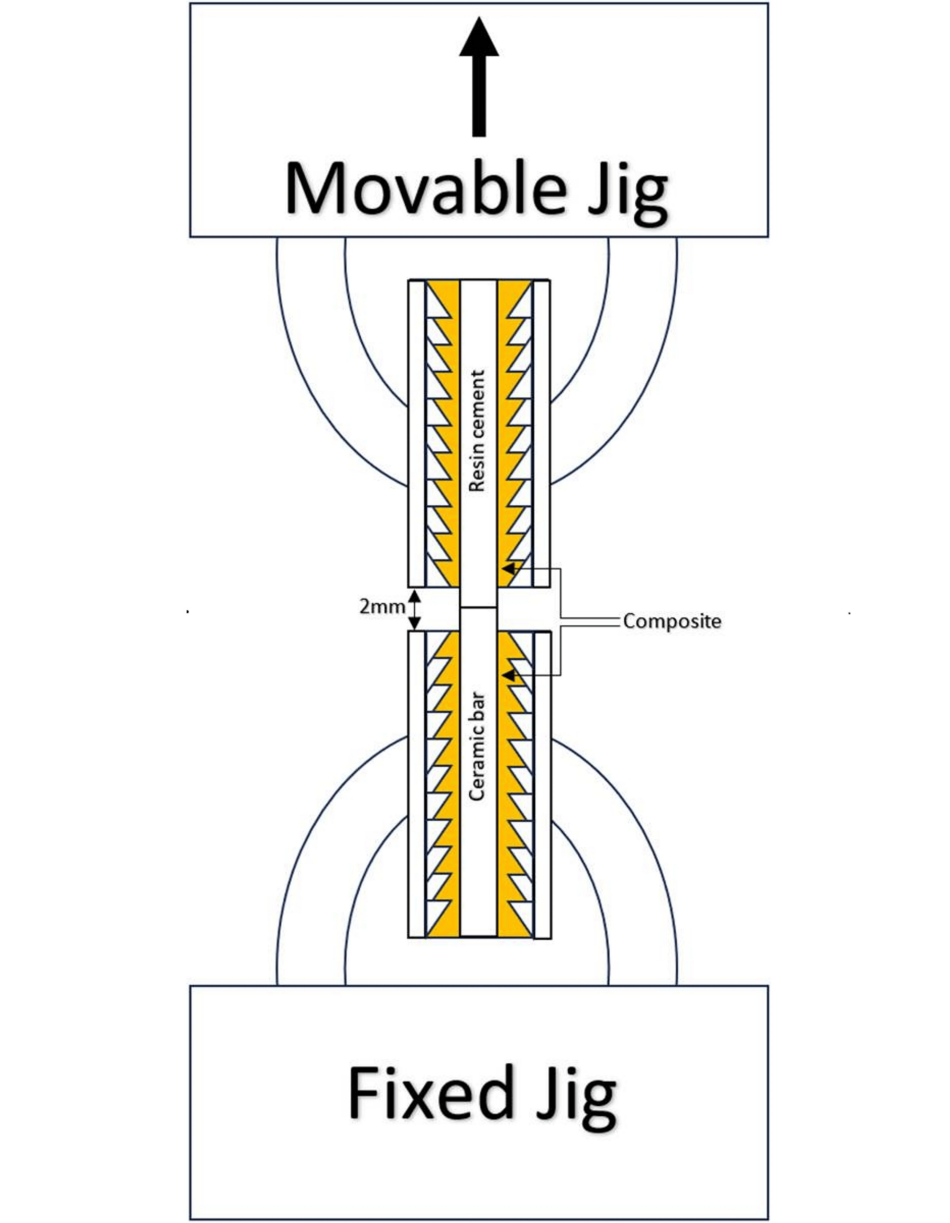
Tensile bond test machine

All groups were subjected to tensile testing (Texture Analyzer EZ TEST, Shimadzu, Osaka, Japan) either before (E0 and L0) or after thermal cycling (E5 and L5). Each specimen was mounted on the jigs of the machine with the cement end upside toward the movable jig. Each jig is composed of two internally serrated parallel plates that can be tightened to fit the specimen accurately. Each side of the specimen (cement and ceramic) was wrapped with composite resin (Filtek Z350 XT, 3M), avoiding the cement-ceramic interface. The plates were tightened, squeezing the composite resin wrap until a close arbitrary distance of about 1 mm between each side of the specimen, and the plates’ serrations were maintained. Pencil markings were made 1 mm farther from each side of the ceramic‐resin interface to ensure midway positioning of the interface and maintain a 2 mm distance between the upper and lower jigs, as shown in Figure 2. The composite wrap was then cured according to the manufacturer’s instructions. Specimens were subjected to tensile force at a 1 mm/min crosshead speed until bond failure. Bond strength was obtained from the following equation: R= F/A, where R is the bond strength (MPa), F is the force applied at the time of failure (Newton), and A is the cross-sectional area of the specimen (mm^2^).

Thermal cycling

E5 and L5 groups underwent an artificial aging process that included 500 thermal cycles between 5 and 55°C. A 30-second dwell time was observed during each thermal cycle, while a 10-second transfer time was permitted between the baths (Mastercycler, Eppendorf AG, Hamburg, Germany).

Statistical analysis

Normality and equal variances were not verified (Shapiro-Wilk’s test and Levene’s test > 0.05). Mean tensile strength was calculated for each group (E0, E5, L0, and L5) using Brown-Forsythe one-way ANOVA and Tamhane’s post hoc tests. Comparisons between groups and identification of outliers were made possible with the use of box and whisker plots. The IBM SPSS Statistics, version 22.0 (IBM Corp., Armonk, NY) was used for statistical analysis.

## Results

**Figure 3:**
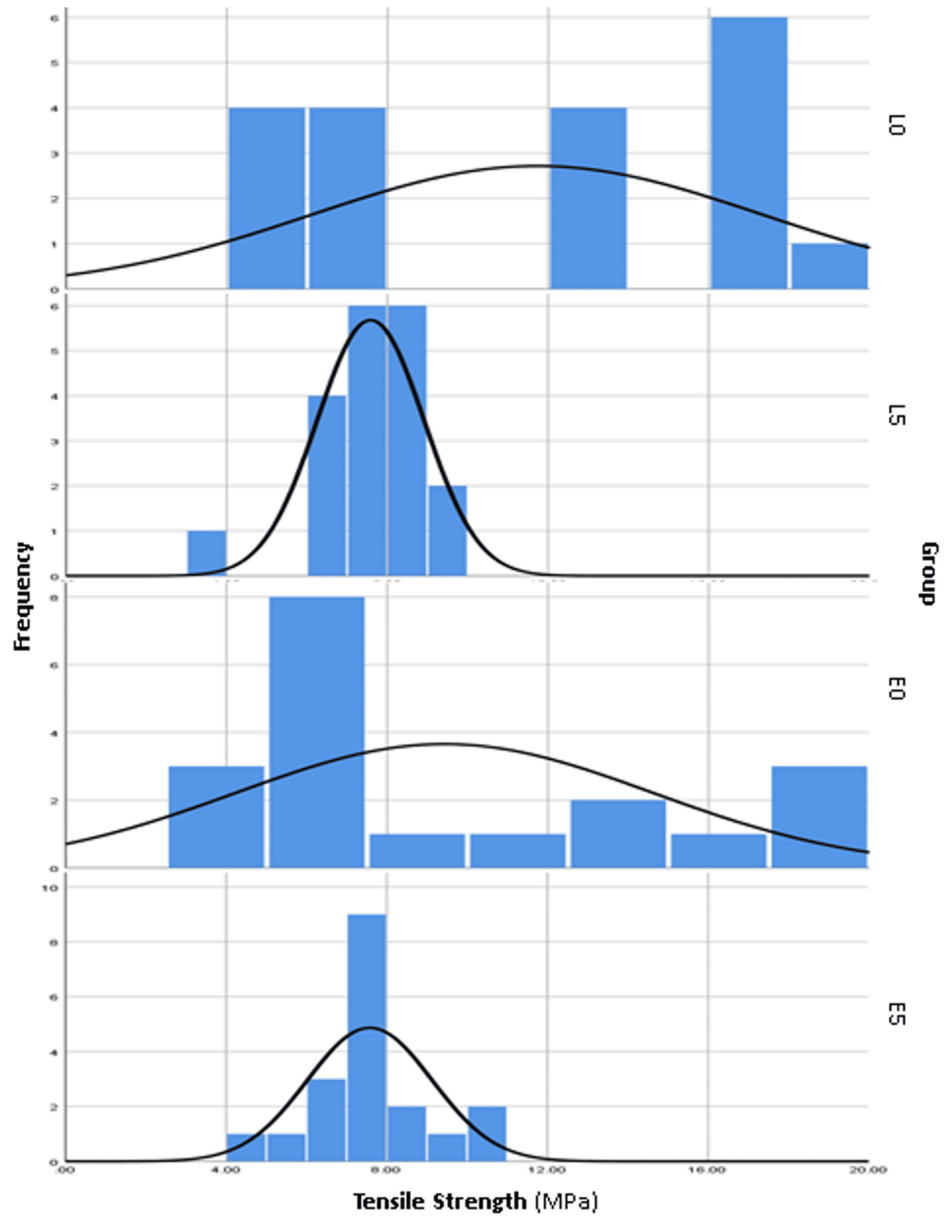
Frequency histogram polygon graph of the TBS stacked by group, comparing the pattern of value distribution between different groups TBS, tensile bond strength

**Figure 4:**
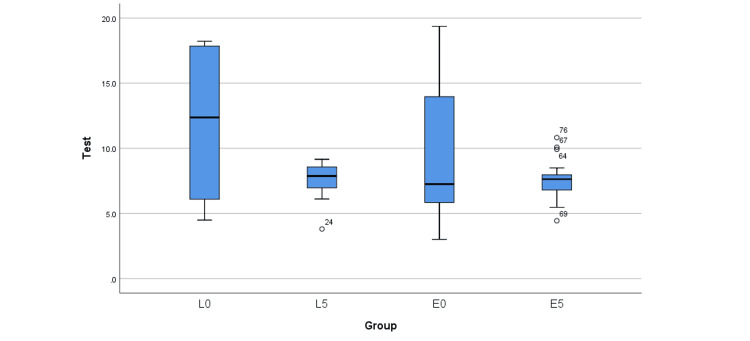
Box and whisker plots comparing the range of values spread between groups Note outliers with E5

The data presented in Table 2 provides a comprehensive overview of the study’s descriptive statistics. Before aging, the mean TBS for Initial LiSi (L0) was 11.7 MPa ± standard deviation (SD) of 5.6, and for IPS E.max CAD (E0), it was 9.4 MPa ± SD 5.2. After aging, these values decreased to 7.6 MPa ± SD 1.3 for Initial LiSi (L5) and 7.6 MPa ± SD 1.6 for IPS E.max CAD (E5). It is evident that both materials experienced a reduction in TBS after aging, with Initial LiSi showing a more pronounced decrease compared to IPS E.max CAD. The range of values (SD) for both materials was significantly wider before aging than after aging. Additionally, Figure 3 reinforces these observations, illustrating broader curves pre-aging (L0 and E0) and narrower curves post-aging (L5 and E5). Furthermore, Table 3 demonstrates the comparisons of the TBS means using Tamhane’s post hoc test, revealing statistically significant mean differences only for the dependent Initial LiSi groups (L0-L5) (p < 0.05). Notably, there were no statistically significant differences between the materials before (L0-E0) or after aging (L5-E5), and no statistically significant difference was found between the dependent IPS E.max CAD groups (E0-E5). The findings presented in Figure 4 through box and whisker plots help visualize the TBS means and values spread between groups along with the identification of outliers. These plots demonstrate an altered behavior of both materials after aging, with IPS E.max CAD exhibiting a greater number of outliers (four outliers) compared to Initial LiSi (one outlier). Lastly, Table 4 highlights the TBS failure modes, indicating mixed (cohesive cement and adhesive) as the predominant failure mode for all groups, followed by adhesive and cohesive modes. Notably, the L5 and E0 groups exhibited the most adhesive failures, while the L0 group had the most mixed failures. Additionally, there were no cohesive failures on the cement side, except in three specimens of the L5 group.

**Table 2:** Descriptive statistics

	N	Mean	Std. deviation	Std. error
L0	19	11.7	5.6	1.3
L5	19	7.6	1.3	0.3
E0	19	9.4	5.2	1.2
E5	19	7.6	1.6	0.4
Total	76	9.1	4.2	0.5

**Table 3:** Summary of analysis *The mean difference is significant at the 0.05 level.

Groups		Mean difference	Sig.
L0	L5	4.13	0.01*
E0	E5	1.85	0.48
L0	E0	2.30	0.28
L5	E5	0.01	1.00

**Table 4:** Modes of failure

Group	Adhesive	Mixed	Cohesive
L0	3	16	0
L5	5	11	3
E0	5	14	0
E5	4	15	0

## Discussion

Since Initial LiSi is still considered new in the dental market, clinical and laboratory evidence is scarce. Each manufacturer of those materials claims its product is superior to the others. Therefore, the authors of this paper have decided to initially compare the tensile strength between both brands at a laboratory level utilizing a commonly used resin cement for the glass-ceramics to investigate which product is superior in that matter. In this study, the only statistically significant difference found was between the pre-aging and post-aging groups of Initial LiSi (L0 and L5) (p < 0.05); thereby, only null hypothesis (ii) was rejected.

The intra-material comparisons showed a significant difference only for Initial LiSi (p < 0.05), which had a relatively higher pre-aging mean (11.7 MPa) than IPS E.max CAD (9.4 MPa); this suggests that thermocycling had a more significant impact on the TBS of Initial LiSi. The microstructure and composition of a lithium disilicate ceramic can influence how its mechanical properties are affected by aging [14]. Previous studies revealed homogenous and smooth surfaces with uniformly distributed crystals in the glass matrix. Different crystal sizes and quantities suggested an explanation for different toughening capabilities between tested specimens [19]. According to the manufacturer, Initial LiSi has a composition of a uniformly distributed submicron of 1-1.5 μm lithium disilicate crystals in a glass matrix. The uniform but smaller crystal size may make Initial LiSi more susceptible to degradation from water and temperature changes compared to IPS E.max CAD. IPS E.max CAD contains larger, 3-6 μm lithium disilicate crystals embedded in a glass matrix (Table 1). The crystal size difference between the materials may explain why Initial LiSi showed a greater drop in the TBS with thermal aging. The uniform but smaller crystals could degrade more easily with temperature fluctuations than the larger crystals of IPS E.max CAD; this is supported by Peumans et al. [11], who found that ceramics with finer crystals were more adversely affected by aging compared to ones with larger grains due to increased surface exposure of crystals to the environment.

The spread of values for both materials was significantly wider pre-aging (pre-aging SDs: 5.2-5.6), as shown in Table 2 and Figure 3. The authors assume that heat could have rearranged the glass matrix on the surfaces of both materials equally, hence the narrow post-aging SDs (1.3-1.6) (Table 1). The variation in the values of the specimens observed before aging could be attributed to the differences in material composition, inherent material heterogeneity, and/or manufacturing flaws, in which significant variation in the values was present. However, the variation narrowed in post-aging as the aging impact is standardized over all specimens. A prior study has investigated the impact of the thermal control of crystallization on the crystal size of the lithium disilicate phase in a lithium disilicate glass-ceramic. The researchers varied the temperature to change the crystal size and examined the effects of this change on the mechanical properties of the glass-ceramic. These findings could explain the variations observed in the values of the tested specimens [20].

Differences in composition, manufacturing techniques, crystal content, and crystallization parameters vary between different lithium disilicate ceramic materials, and this is reflected not only in the different microstructures of these materials but also in their mechanical and clinical performance. These factors interact in the process of establishing a strong bond at the ceramic-cement interface. It has been reported that IPS E.max CAD has higher lithium disilicate content than Initial LiSi, which could lead to the high fracture resistance of IPS E.max CAD compared to Initial LiSi’s [21]. In addition, IPS E.max CAD undergoes a heating cycle after milling for complete crystallization, unlike Initial LiSi, which is provided in its complete crystallized form, so microcracks present in blocks or that may have occurred during milling might have disappeared after heat exposure of IPS E.max CAD [22]. This implies that heat cycles of IPS E.max CAD could reduce post-milling surface flaws if present, presumably enhancing a healthier ceramic-cement interface. Moreover, the densely distributed lithium disilicate crystals in Initial LiSi and IPS E.max CAD can slow crack growth, which may, in turn, contribute to their higher flexural strengths [21]. Certain research has indicated a clear link between the bond strength output and the elastic modulus of the bonding substrate; this could participate in the justification of the relatively higher pre-aging TBS of Initial LiSi, as Initial LiSi possesses a narrower difference of the elastic modulus with bonding substrate compared to IPS E.max CAD [23,24]. All these factors can introduce variations in material characteristics and microstructural features.

The decrease in bond strength after aging can be attributed to the effects of fatigue and environmental factors on ceramic restorations. Factors such as thermal cycling, exposure to the oral environment, and dynamic stresses can lead to fatigue and degradation of the material, resulting in decreased bond strength [25]. The results showed that all tested specimens demonstrated a decrease in bond strength after aging, with Initial LiSi specimens demonstrating a more significant drop than the other specimens. As previously explained, this behavior may be attributed to the chemical interactions and degradation processes specific to Initial LiSi.

IPS E.max CAD showed many outliers compared to Initial LiSi post-aging (Figure 4); however, there was no statistically significant difference between the post-aging means of both materials. The more remarkable number of outliers in the IPS E.max CAD group could be related to the size of lithium disilicate crystals, which can affect the overall material properties. For example, a study by Yao et al. [2] reported that smaller crystal sizes and more uniform distribution of crystals resulted in higher tensile strength values. On the other hand, Alhomuod et al. [26] have evaluated the effect of microstructure on the shear bond strength of four different lithium disilicate ceramics and found that bond strength to CAD/CAM lithium disilicate glass-ceramic materials is significantly affected by their microstructure, surface treatment, and aging. The loose distribution of the lithium disilicate crystals in the glassy matrix may contribute to more cohesive failures. Nonetheless, the authors found that the size of the lithium disilicate crystals in all groups does not appear to interfere with the resin permeation into the etched microstructure of the ceramics. 

Most failure modes were mixed (cohesive cement and adhesive), which implies enhanced bond strength for all groups (Table 3). The cement side had no cohesive failures except in the Initial LiSi specimens, showing relative superior stability at the bond interface. Initial LiSi is superior in the consistency of its figures, which implies more predictability of the bond outcome. The predominance of mixed failure mode is attributed to the material’s fracture behavior and microstructural characteristics. Lithium disilicate ceramics exhibit a combination of brittle and tough fracture behavior. Pospiech [3] explored the bonding procedure in ceramic restorations and emphasized the importance of achieving a solid and durable bond between the ceramic and the adhesive. The author highlighted that a successful bond should withstand stresses generated by polymerization and shrinkage of the bonding agent. A mixed mode of failure was considered indicative of a successful bond.

Future trials are encouraged to increase the number of cycles and explore different thermocycling protocols to better mimic clinical conditions. Nevertheless, the findings of this study provide valuable insights for clinicians in the wise selection of appropriate lithium disilicate glass-ceramic material when higher retention is required for specific clinical scenarios. Medium- and long-term clinical trials are encouraged to correlate the relevance between the laboratory outcomes and the clinical durability of the bond strength of restorations fabricated from both materials.

The limitations of this study should be approached thoughtfully. Firstly, it’s important to acknowledge that invitro studies have limited relevance in determining clinical durability due to potential differences in real-world applications. Additionally, the ceramic specimens were not bonded to teeth, potentially not simulating clinical settings. Moreover, the length of the specimens may have influenced the distribution of tensile force around the ceramic-cement interface, impacting the study’s results. Furthermore, the lack of standardized force application for holding the specimens is a notable limitation, as an arbitrary application of force may introduce variability. These limitations underscore the need for careful interpretation of the study’s outcomes and suggest areas for potential improvement in future research.

## Conclusions

After conducting a thorough analysis of the experiment’s results, the authors drew several notable conclusions. Initial LiSi exhibited the highest pre-aging mean, and thermocycling had a more pronounced impact on Initial LiSi compared to IPS E.max CAD. Additionally, thermocycling had a significant effect on the consistency of values for IPS E.max CAD and Initial LiSi. Importantly, there were no statistically significant differences between the pre- and post-aging bond strengths of IPS E.max CAD and Initial LiSi. The authors conclude that IPS E.max CAD bond strength is relatively more predictable than that of Initial LiSi.

To better understand the impact of thermocycling on bond stability, additional research is necessary. Analyzing the microstructure before and after aging may provide insights into the notable greater decrease in bond strength observed in the Initial LiSi specimens.
